# Diabetes

**Published:** 1905-10-21

**Authors:** 


### DIABETES.

**The Source of the Sugar in Diabetes.**—It is an established fact that sugar may be formed from either proteid, carbohydrate, or fat; the view that in diabetes the liver forms and passes into the general circulation an excessive amount of sugar is questioned by Pavy,[1] who has failed to find any excess of sugar in the blood of the hepatic vein. Nor does he find that arterial blood generally contains more sugar than venous blood, which it should do if sugar is carried as such to the muscles for use. By a series of very exhaustive experiments Pavy has shown that any excess of sugar in the blood is immediately excreted by the kidneys, and seeing that sugar must be carried in some way to the seat of its oxidation, he has concluded that it is present in the blood incorporated with the proteid molecule; he has also succeeded in obtaining a carbohydrate group from various kinds of proteid substances. In further support of this view he instances the assimilation of sugar into the proteid molecule, which has been proved to take place in the yeast plant. Pavy regards the lymphocytes as carriers both of the proteid and carbohydrate of food, the former substance being utilised for tissue production and the latter as a source of energy. In support of this he adduces (1) the well-established fact of digestion lymphocytosis, (2) the crowding of the intestinal villi with lymphocytes during the period of active absorption, (3) the increased urea production which occurs as the lymphocytosis passes off. Glycogen he regards as merely a storage product analogous to fat. Bosanquet[2] while holding similar views as to the position of glycogen in the animal economy, and believing that only slight and transient glycosuria can be derived from its conversion into sugar in excess of the needs of the organism, is inclined to view fat as the principal source of sugar in diabetes. He does not believe that in diabetes sugar is directly produced from food, but that it is formed by faulty metabolism in the body tissues. The increased nitrogen elimination which corresponds closely to the sugar elimination, he does not consider to prove that the sugar is derived from proteid. He points out that acetone excretion varies in exactly the same ratio, and that the increased proteid destruction may merely be due to acid intoxication. He quotes various experiments which show that sugar may be formed from fat both within and without the body; and notes that in the katabolism of fat sugar may be regarded as coming from the glycerin by a fairly simple chemical process, and the acetone bodies from the fatty acid. The further points he adduces, such as the occurrence of liphæmia and a fatty liver, the relationship between diabetes and obesity, and certain experimental observations all tend to show that some disturbance of normal fat metabolism occurs in diabetes.

**The Varieties of Sugar in Diabetes.**—Although glucose is the chief sugar found in the blood and urine both under normal conditions and in disease, Pavy has shown that other forms of sugar are also present. The urine in healthy animals normally contains sugars of a lower reducing power than the blood, the amount of reducing power shown by the urine depending to a great extent on its concentration—*i.e.* urines of high specific gravity sometimes show (apart from diabetes) a remarkably high reducing power over cupric oxide. If, however, the sugar in such an urine be hydrolysed with an acid, the reducing power is heightened to a very considerable extent, which does not occur in diabetes, owing to the fact that in this disease glucose (dextrose) is the principal component of the sugar excreted and has a higher reducing power than any other form. It is thus of importance in any case of glycosuria to determine the kind of sugar excreted. Cammidge[3] has drawn attention to the delicacy of the phenylhydrazin test in this connection. No doubt much valuable information may be obtained by this method, and light thrown upon the nature of anomalous carbohydrate metabolism, but the necessary technique is somewhat complicated. To distinguish the different osazones formed, Cammidge enumerates five tests: (1) the rate of osazone formation; (2) the microscopical characters of the crystals; (3) their solubilities in various reagents; (4) the melting-points of the purified products; (5) their percentage content of nitrogen. For the differentiation of dextrose and lævulose however, further tests, such as the rotary power, must be employed.

**Non-Saccharine Reducing Agents.**—It has long been known that many substances may occur in urine which are capable of reducing cupric oxide, but are not sugars. Of these, creatin and its allies (hippuric and uric acids) form no osazone crystals, and thus may be distinguished by the phenylhydrazin test. On the other hand, substances are found which both reduce cupric oxide and form osazone, such as glycuronic acid. This substance, which occurs combined with various bases and in very small quantities in healthy urine, is increased considerably under various conditions. Cammidge states that the ingestion of cane-sugar, the presence of putrefactive changes in the intestine, chloroform anæsthesia, and various drugs (such as camphor, chloral, salicylic acid, thymol, turpentine), all lead to increased excretion of glycuronic acid. There are, however, other substances set free in the urine by boiling with dilute acids which form osazone crystals distinguishable from those of glycuronic acid. It was on this fact that Cammidge founded his well-known test for pancreatic disease. This test is still under investigation, but Cammidge's conclusion as to the value of phenylhydrazin as a delicate reagent for inquiring into the variations of the urine in disease is certainly not likely to be disputed.

**Points in the Diagnosis and Treatment.**—A curious observation has recently been tested by Williamson[4] on what he terms the "vibrating sensation." This is the sensation normally produced when a vibrating tuning-fork is placed over certain bony prominences of the body, such as the styloid process of the ulna. The vibration under normal conditions is felt to extend throughout the whole length of the bone. The observation was originally made in Germany, and has been confirmed by several authorities. It is said to be always present in health, but absent in certain nervous diseases and in diabetes mellitus. Williamson's results confirm those of other observers. He finds, however, that the kind of tuning-fork selected is of importance, as the sensation imparted varies, and that the part of the body chosen for the test should be noted, as here, too, individual variations exist. The significance of this phenomenon does not seem clear, but from a diagnostic point of view it would appear to be more useful in early cases of nervous disease than in diabetes, for though the "vibrating sensation" is present in diabetes insipidus and absent when sugar is present in the urine, there is no distinction found between true diabetes mellitus and various forms of non-diabetic glycosuria.

In the treatment of diabetes preparations of yeast have recently been advised. A test of this substance has recently been undertaken[5] which shows that it appears, when given by the mouth, to withstand the action of the digestive juices, and, in mild cases, to reduce to a certain extent the amount of sugar excreted. More than this cannot be said for it, though it has a much more marked influence on boils and carbuncles, and may thus be of value in the treatment of one of the complications of diabetes mellitus. Various "medicinal" preparations of yeast are on the market, but, apart from æsthetic considerations, they do not appear to possess any advantage over ordinary brewers' yeast.

[1] Lancet, June 24, 1905; July 1, 1905; August 5, 1905. [2] Ibid., April 8, 1905; April 15, 1905; April 22, 1905. [3] Ibid., July 1, 1905, p. 14. [4] Ibid., April 1, 1905, p. 855. [5] Brit. Med. Jour., April 12, 1905, p. 352.

